# Human T-cell lymphotropic virus (HTLV)-associated encephalopathy: an under-recognised cause of acute encephalitis? Case series and literature review

**DOI:** 10.1007/s00415-018-8777-z

**Published:** 2018-02-08

**Authors:** Ania A Crawshaw, Divya Dhasmana, Brynmor Jones, Carolyn M Gabriel, Steve Sturman, Nicholas W S Davies, Graham P Taylor

**Affiliations:** 1 National Centre for Human Retrovirology, Imperial College Healthcare NHS Trust, Praed Street, London, W2 1NY UK; 2 Department of Neuroradiology, Imperial College Healthcare NHS Trust, Praed Street, London, W2 1NY UK; 3 Department of Neurology, Imperial College Healthcare NHS Trust, Praed Street, London, W2 1NY UK; 4 Department of Neurology, University Hospitals Birmingham NHS Trust, Edgbaston, Birmingham, B15 2TH UK; 5 Department of Neurology, Chelsea and Westminster Hospital NHS Trust, 369 Fulham Rd, Chelsea, London, SW10 9NH UK

**Keywords:** HTLV-1, Encephalopathy, Encephalitis, Corticosteroids, Case series, Review

## Abstract

Human T-cell lymphotropic virus (HTLV)-1-associated myelopathy (HAM) is well described. Clinical features are predominantly consistent with cord pathology, though imaging and autopsy studies also demonstrate brain inflammation. In general, this is subclinical; however, six cases have previously been reported of encephalopathy in HTLV-1-infected patients, without alternative identified aetiology. We describe three further cases of encephalitis in the UK HAM cohort (*n* = 142), whereas the annual incidence of acute encephalitis in the general population is 0.07–12.6 per 100,000. Clinical features included reduced consciousness, fever/hypothermia, headaches, seizures, and focal neurology. Investigation showed: raised CSF protein; pleocytosis; raised CSF:peripheral blood mononuclear cell HTLV-1 proviral load ratio; and MRI either normal or showing white matter changes in brain and cord. Four of the six previous case reports of encephalopathy in HTLV-infected patients also had HAM. Histopathology, reported in three, showed perivascular predominantly CD8+ lymphocytic infiltrates in the brain. One had cerebral demyelination, and all had cord demyelination. We have reviewed the existing six cases in the literature, together with our three new cases. In all seven with HAM, the spastic paraparesis deteriorated sub-acutely preceding encephalitis. Eight of the nine were female, and four of the seven treated with steroids improved. We propose that HTLV-associated encephalopathy may be part of the spectrum of HTLV-1-induced central nervous system disease.

**Electronic supplementary material:**

The online version of this article (10.1007/s00415-018-8777-z) contains supplementary material, which is available to authorized users.

## Introduction

Encephalitis refers to inflammation of brain parenchyma and should be suspected clinically in the presence of fever, headache, and altered consciousness [1]. It may occur as a direct effect of infection or be immune-mediated, as in acute disseminated encephalomyelitis (ADEM) or antibody-associated limbic encephalitis [2]. In 37% of acute encephalitis cases, the cause is not identified [3].

Human T-cell lymphotropic virus type 1 (HTLV-1) is a retrovirus which causes HTLV-1-associated myelopathy (HAM) in 0.25–3.7% of infected individuals [4]. Asymptomatic individuals who have high peripheral blood mononuclear cell (PBMC) HTLV-1 proviral load (> 1%) are at risk of developing myelopathy. High HTLV-1 CSF:PBMC ratio is a diagnostic feature of HAM [5].

Pathological and imaging studies suggest both the brain and cord can become inflamed in HTLV-1 infection. Histopathological analyses in HAM at autopsy have demonstrated perivascular predominantly CD8+ inflammatory infiltrates throughout the central nervous system (CNS) in the presence of active-chronic spinal cord disease, compared to inactive-chronic spinal cord disease, where inflammation was noted in the spinal cord only [6]. Leucoencephalopathy is often present on brain magnetic resonance imaging (MRI), to a greater degree than in age-matched controls [7]. Positron-emission tomography (PET) assessing microglial activation has also demonstrated significant neuroinflammation in the brains of HAM patients and, to a lesser degree, asymptomatic HTLV-1 carriers [8]. Although the most common clinical manifestations of HTLV-1 neuroinflammation are restricted to the thoracic spinal cord, the reported presence of inflammation in the brain should prompt physicians to be vigilant for clinical signs and symptoms indicative of intracranial pathology.

The national HTLV-1 cohort in England includes 142 patients with HAM. Three have had encephalitis—an estimated incidence of 1 per 359 person-years follow-up. This equates to a rate of 278/100,000 person-years compared with the reported annual incidence of encephalitis in England of 5.23 cases per 100,000 population [9]. Here, we describe these three patients with HTLV-associated encephalitis, and review the existing reports of HTLV-1-infected patients who became encephalopathic.

## Methods

A retrospective case note review was undertaken of all patients with HAM in our UK cohort who developed encephalitis (1995–2017).

The literature was reviewed using PubMed. The search enquiry was (((HTLV) OR (HTLV-1) OR (HTLV-I)) AND ((encephalitis) OR (encephalopathy) OR (encephal*)). Included in the review are reported cases of acute- or sub-acute-onset encephalopathy in patients with HTLV-1 infection. Cases where another more likely aetiology of encephalopathy was present were excluded from the discussion. The literature search was last updated in October 2017.

## Retrospective case series

### Patient A

A 35-year-old Caucasian female had an episode of encephalitis 6 years after diagnosis of HTLV-1 infection, 2 years after onset of HAM. Over 2 months, her mobility worsened and she was admitted electively for investigation. On examination, she had low-grade fever and spastic paraparesis with hyperreflexia in all four limbs, though no meningism. She could not stand unaided, while previously had mobilised with a stick. Over the next 2 days, she became pyrexial at 39 °C, but remained haemodynamically stable. She then became drowsy and disorientated and had two generalised seizures. Despite broad-spectrum antibiotics (ampicillin, cefuroxime, and gentamicin) and IV aciclovir, her GCS fell acutely to 5 before improving, though she had ongoing confusion with visual hallucinations and severe headache.

Investigation revealed mild neutrophilia with elevated C-reactive protein of 32 (normal < 5 mg/L) and alanine aminotransferase 166 (0–40 IU/L). Lumbar puncture (LP) 6 h after beginning antibiotics showed opening pressure 24 cmH_2_0 and mild pleocytosis (9 predominantly lymphocytes per mm^3^). CSF protein was raised at 2.33 g/L. No organisms were identified on extended cultures. Other investigations including MR brain were unremarkable (see Supplementary Online Table). This event predated the availability of HTLV-1 proviral load measurement. Coliform bacteria grew on urine culture (sensitive to her prescribed antibiotics).

Four days later, she improved clinically, becoming alert and orientated, but with no recall of recent days. Cognitive problems (amnestic and language) persisted for several months before completely resolving.

### Patient B

A 52-year-old Caucasian female was wheelchair dependent at initial diagnosis of HTLV-1 infection and HAM. She had recurrent HTLV-associated uveitis and a 40 pack-year smoking history. Six years after initial diagnosis, she enrolled in a clinical trial of infliximab, however, after two doses, developed a petechial rash and severe headache so was withdrawn from the trial. The headache and rash resolved over 2 months. She then developed *Haemophilus influenzae* pneumonia, treated with three courses of oral antibiotics over a month.

Five months after infliximab withdrawal, her mobility deteriorated and she could no longer transfer independently. MRI brain and cervical spine revealed cervical spondylosis but nil else of note. Eight months following infliximab withdrawal, an outpatient course of 3-day pulsed IV methylprednisolone led to a marked improvement—she could again transfer independently and straight leg raise bilaterally. The improvement was short-lived. After 1 month, she developed worsening headaches.

Ten months after infliximab cessation, she developed partial left sixth and third nerve palsies with anisocoria and blurred vision. In the upper limbs, there was bilateral intention tremor and left dysdiadochokinesis. In the lower limbs, she had longstanding spasticity, but now had only a flicker of movement. She was apyrexial, haemodynamically stable and oriented.

**Fig. 1:**
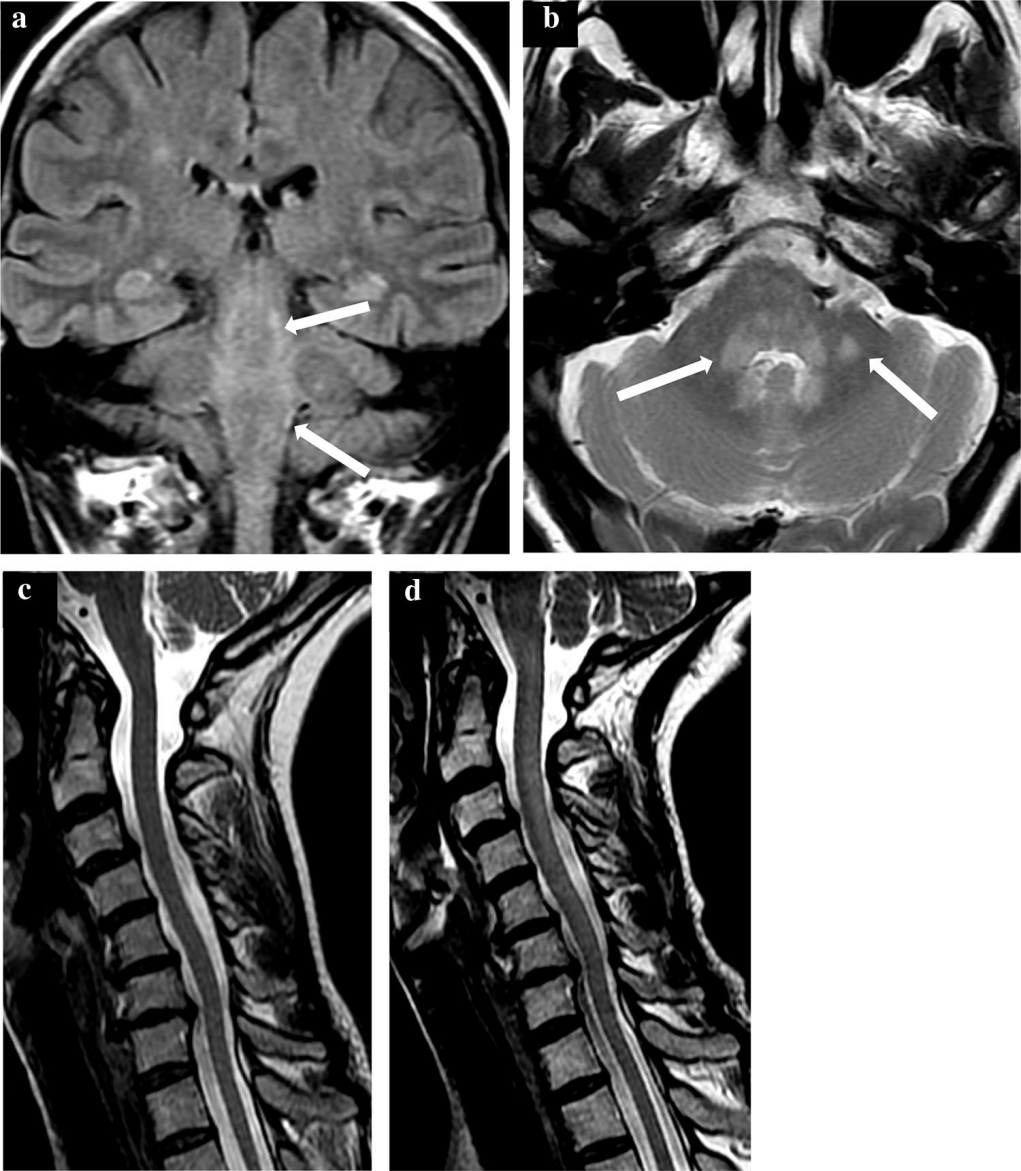
**a**, **b** Patient B MRI brain imaging during first episode of encephalitis. **a** Coronal FLAIR and **b** axial T2 FSE. Widespread infratentorial parenchymal signal abnormality particularly involving the dorsal brainstem with extension into the cervical spinal cord. There was minimal mass effect and patchy gadolinium enhancement. In addition, there was scattered involvement of the fronto-parietal white matter (not shown). Appearances are in keeping with an acute inflammatory process, whether infective or para-infective. Follow-up imaging 2 months later showed near resolution of the brainstem changes. **c** Patient B MRI cord imaging 5 months prior to encephalitis admission showing normal cervical cord appearances. **d** MRI during first encephalitis episode showing subtle ill-defined long segment signal abnormality throughout the cervical cord which is also slightly swollen. Appearances are those of a long segment myelitis. There was no pathological enhancement

Repeat MRI showed several new scattered focal T2-weighted lesions mainly in the peripheral white matter of both cerebral hemispheres, most numerous in the frontal lobes. Compared to imaging 5 months previously, there was new diffuse signal change in the brainstem, particularly the dorsal pons and medulla oblongata, as well as the adjacent middle cerebellar peduncles, cerebellar white matter, and cervical cord (Fig. 1a, b). The upper cervical cord was oedematous (Fig. 1d). The imaging findings were not in keeping with demyelinating disease. LP revealed normal opening pressure and 20 monocytes/mm^3^ CSF. Protein was slightly elevated at 0.67 g/L. Normal/negative investigation results are summarised in the supplementary online table. During this episode, HTLV-1 CSF proviral load was 120 per 100 CSF cells (120%), in contrast to 17.9% in PBMCs (Fig. 4a). She remained cardiovascularly stable throughout admission and blood cultures were sterile. *Haemophilus influenzae*, present on sputum culture, was treated with oral co-amoxiclav.

After 19 days in hospital, no additional explanation for her neurological symptoms had been identified. In the absence of contraindications, 3-day pulsed 1 g intravenous methylprednisolone was given. Her headaches and diplopia improved within days. Seven weeks after admission, MRI brain and cord showed marked resolution of the white matter changes; only those in the left cerebellar peduncle remained.

Eight months after the initial encephalitis admission, patient B gave a 6-month history of mild memory problems, intermittent mild diplopia, and recurrence of frontal headaches. She had been admitted electively for debridement of a sacral decubitus ulcer. Five days following this procedure, the left sixth nerve palsy recurred. Her conscious level deteriorated rapidly, necessitating intubation. Temperature was 39 °C, and she was started on vancomycin, meropenem, metronidazole, and clindamycin in the context of previously positive MRSA skin swabs.

**Fig. 2:**
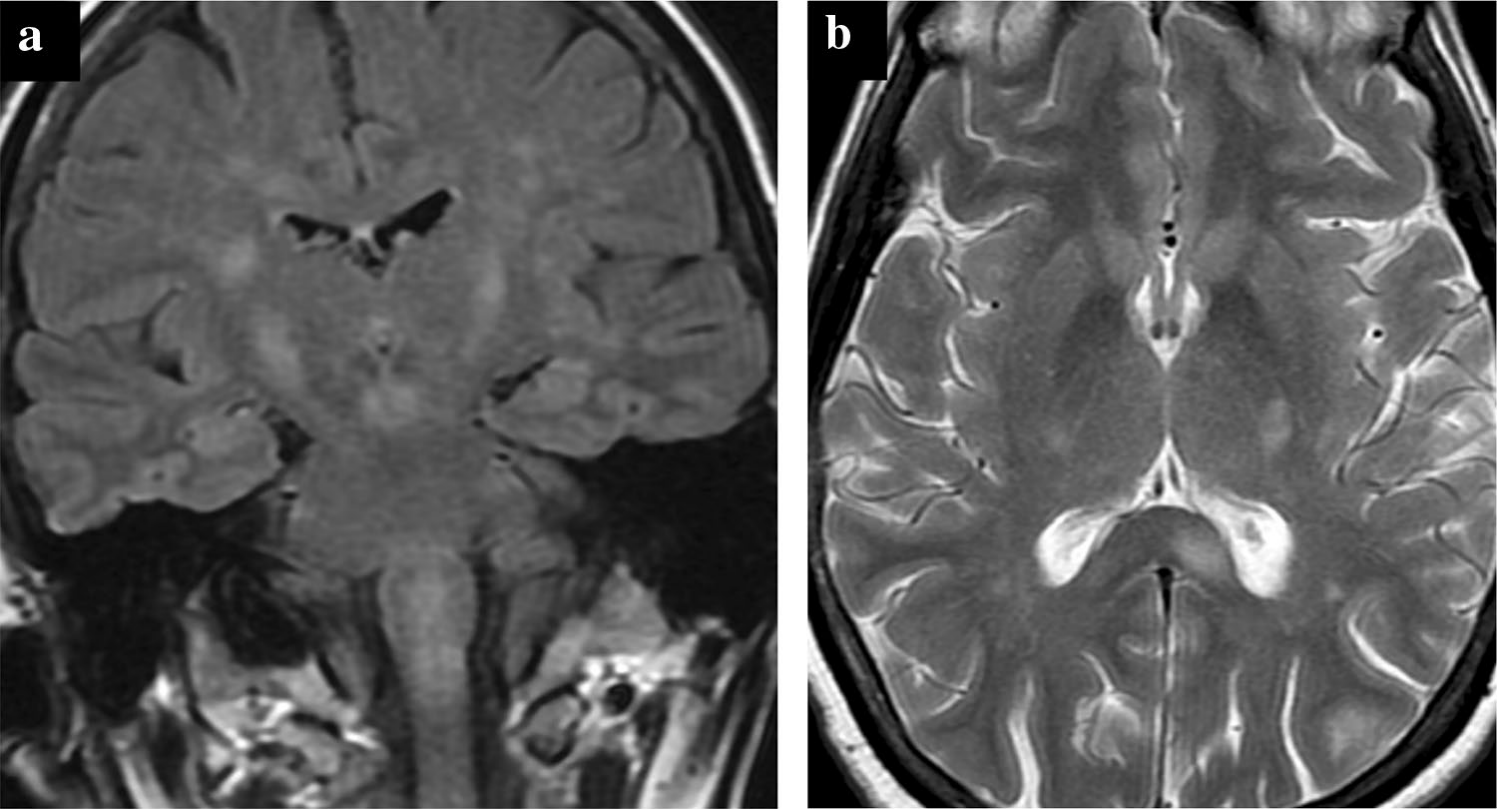
**a**, **b** Patient B MRI brain imaging during second episode of encephalitis. **a** Coronal FLAIR and **b** axial T2 FSE. New patchy signal change in the fronto-parietal white matter, splenium of the corpus callosum, cortico-spinal tracts and recrudescence of the diffuse brain stem signal change seen during the previous encephalitic episode

MRI showed recurrence of the diffuse white matter changes in the brainstem as well as the centrum semiovale, corpus callosum, cortico-spinal tracts, and cerebellar peduncles, though the cord white matter on this occasion was spared (Fig. 2a, b). LP was contraindicated due to proximity of the sacral ulcer. When she did not improve neurologically after 1 week, high-dose methylprednisolone was again administered. A few days later, she improved and was extubated before being discharged home. Follow-up imaging showed persistence of some supratentorial focal T2 hyperintensities, but near resolution of the diffuse brainstem and cerebellar abnormalities.

Although she had no further encephalopathic episodes, HAM symptoms progressed and she died 2 years later.

### Patient C

A 43-year-old diabetic Afro-Caribbean female was diagnosed with HTLV-1 infection during investigation for spastic paraparesis. Four months later, she developed disseminated varicella zoster virus (VZV) infection with multi-dermatomal skin lesions and encephalitis, alongside worsening myelopathy. VZV was detected on CSF PCR, with 26 white cells/mm^3^ and protein 0.6 g/L. MR brain showed no abnormality. She improved clinically with IV valaciclovir, but relapsed with recurrent encephalitis on its withdrawal. She was maintained long term on twice daily 500-mg valaciclovir thereafter.

One year later, her mobility sub-acutely deteriorated and she developed hypothermia (31–33 °C) followed by acute reduction in consciousness without new focal neurology. She had no rash. On LP, 32 white cells/mm^3^ were seen, and protein was 0.7 g/L. VZV was not detected in the CSF.

**Fig. 3:**
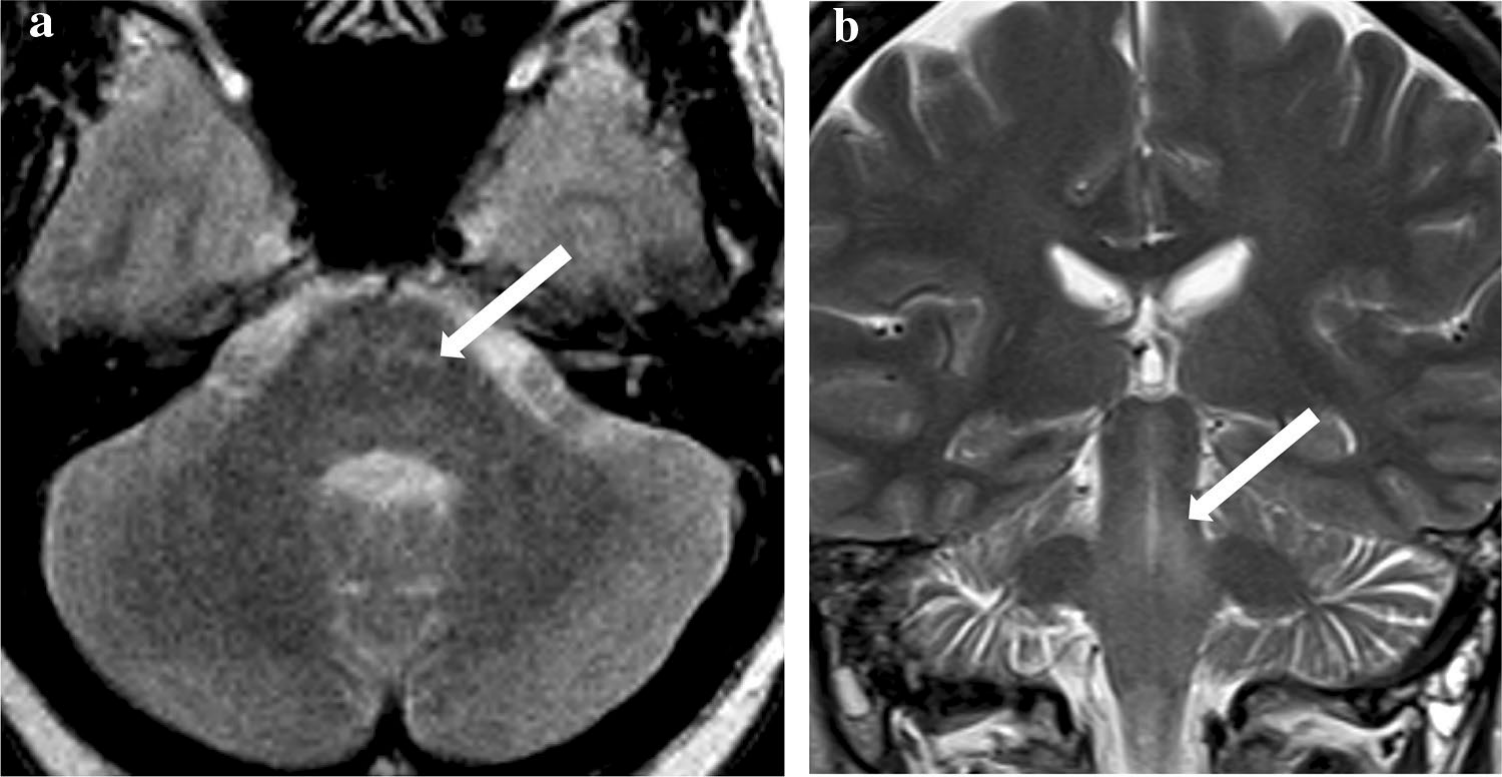
**a** Patient C MRI brain during third episode of HTLV encephalitis. Ill-defined T2w signal hyperintensity in the basis pontis. **b** Patient C brain MRI brain during fourth episode of HTLV encephalitis (while on ciclosporin). Coronal T2w sequence. Ill-defined T2 signal hyperintensity in the midbrain and pontine tegmentum. No swelling or pathological enhancement. Previous area of signal change now normalised

**Fig. 4:**
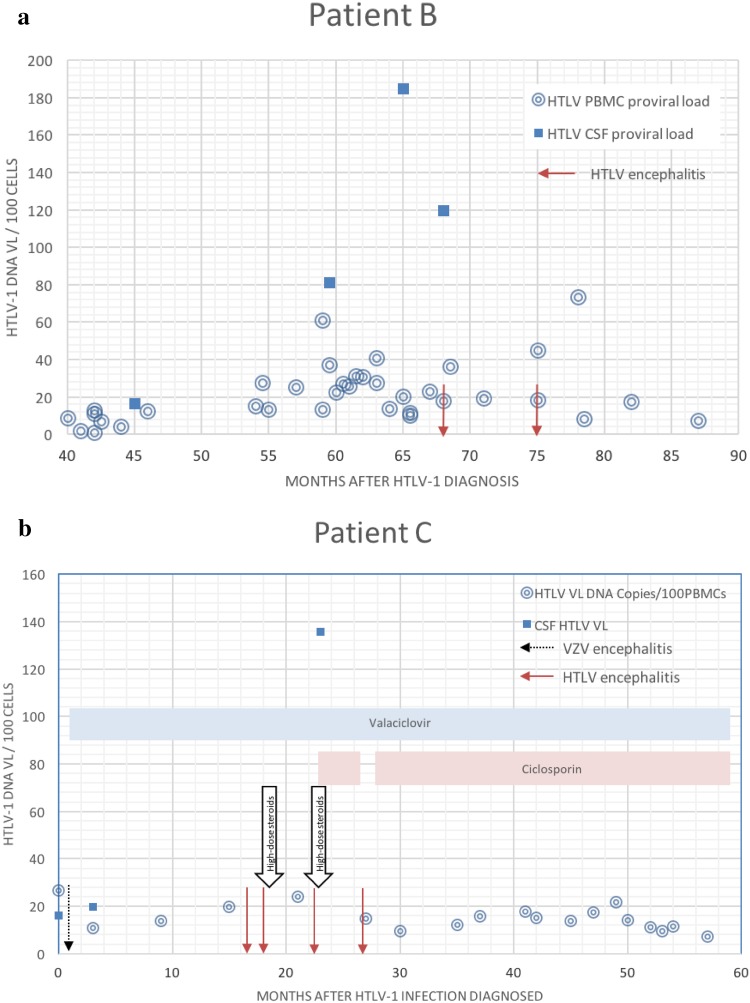
**a**, **b** Variation in HTLV proviral load in PBMC and CSF in patients B and C, respectively. Episodes of encephalitis are marked with arrows

During three subsequent similar episodes, she was also initially hypothermic (31–33 °C). Two episodes were preceded by 2 weeks of global aphasia and all were associated with reduced consciousness. VZV was not identified by CSF PCR though CSF showed pleocytosis (6–32 cells/mm^3^ CSF) in all but the penultimate episode. CSF protein ranged from 0.53 to 0.85 g/L. Investigation did not reveal another recognised cause for encephalitis (Supplementary Table). No MR abnormalities were evident during the first two episodes; however, during the third, MR imaging showed ill-defined T2-weighted pontine hyperintensities (Fig. 3). Figure 4b shows the variation in HTLV proviral load with time and episodes of encephalitis. CSF HTLV proviral load was higher than in PBMC.

She received high-dose dexamethasone after episodes two and three of VZV-negative encephalitis, each time with rapid clinical recovery after 2–3 days. Ciclosporin was then initiated at a dose of 2.5 mg/kg/day. Nine weeks later, she developed treatment-refractory hyperkalaemia secondary to ciclosporin toxicity. Five days after ciclosporin withdrawal, she acutely deteriorated with hypothermia and reduced conscious level. LP revealed acellular CSF, in contrast to the previous episodes, though protein was 0.85 g/L. High-dose steroids led to rapid recovery within 2 days. Ciclosporin was restarted and successfully continued without further episodes for 4 years.

Her final encephalitis relapse occurred, while on ciclosporin, this time with initial hypothermia, diplopia, and acutely reduced conscious level. She again recovered on high-dose steroids. She was initiated on long-term mycophenolate mofetil thereafter. She had ongoing thermal dysregulation, putatively secondary to hypothalamic damage post-encephalitis. She died 1 year later with an aspiration pneumonia.

### Case series summary

In these three women with HAM, no recognised aetiological agent was identified for their eight episodes of encephalitis. In each case, a preceding or concurrent infection with another organism was present: coliform urine infection, Haemophilus pneumonia, and VZV. None of these in isolation explain the encephalitic presentations; however, it may be that these concurrent illnesses acted as a trigger for worsening HAM symptoms followed by HTLV-associated encephalitis. MRI findings in two patients showed reversible patchy white matter changes in the brainstem. Although CNS demyelination [10] and progressive multifocal leukoencephalopathy [11, 12] have been reported during treatment with infliximab, the imaging findings in case B were not typical of either, John Cunningham (JC) virus was not detected by PCR and the event occurred 10 months after the last of two doses of infliximab.

When assessed, CSF:PBMC HTLV proviral load ratios during episodes of encephalitis were high, apart from during the VZV-positive encephalitis experienced by patient C when the ratio was less than 1. Patients A and B also had evidence of more widespread HTLV-associated inflammation, with alveolitis and uveitis, respectively. These findings, alongside the reversible MRI white matter changes in patients B and C and their responsiveness to immunosuppression in the absence of an alternative aetiology could support the notion that HTLV infection can cause encephalitis. Patients deteriorating with HAM are also known to respond to steroid treatment [13, 14].

## Results of the literature review

346 articles were retrieved through the initial PubMed enquiry. Of the reported cases with both HTLV-1 infection and encephalopathy, excluded from discussion were those with co-existing: haematological malignancy (*n* = 15); paraneoplastic limbic encephalitis associated with a breast tumour (*n* = 1); progressive multifocal leukoencephalopathy (*n* = 6); tuberculosis (*n* = 1); strongyloides hyperinfection (*n* = 1); toxoplasmosis (*n* = 1); and HIV (*n* = 1). We also identified two case reports describing women with concurrent neuromyelitis optica (NMO) [15, 16].

Six case reports described HTLV-1-infected patients with encephalopathy who had no other identified explanation for their encephalopathy (Table 1). Four of these had HAM [17–20], while two had no evidence of myelopathy [21, 22].

**Table 1:** Summary of six existing case reports of HTLV-associated encephalopathy

References	Araga et al. [21]	Iwata et al. [22]	Smith et al. [17]	Tachi et al. [19]	Tateyama et al. [20]	Puccioni-Sohler et al. [18]
Age/sex	52, F	49, M	73, F	13, F	65, F	41, F
Ethnicity	Japanese	Japanese	African–American	Japanese	Japanese	Brazilian
Clinical features	Meningoencephalitis.	Encephalopathy and ataxia	Encephalopathy with seizures	Encephalo-myelopathy with myoclonic seizures	Encephalo-myelo-neuropathy	Rapid-onset myelopathy then encephalopathy with seizures
Myelopathy	No	No	Yes	Yes	Yes	Yes
Time after HAM onset	**–**	–	24 years	Months	~ 1 month	~1 month
CSF cells	176–1360 white cells (initially monocytes, then mostly lymphocytes)	1 lymphocyte	Acellular	33 white cells (lobulated nuclei)	1 white cell	7–116 white cells (first mostly lymphocytes, then mixed with neutrophils)
Other abnormal CSF features	IgG index 0.62, OP 36 cmH_2_O			Protein S3	Protein 95, oligoclonal bands	Protein 69–83, IgG index 0.7–1.2
HTLV-1 blood	Antibody 1:4096	Antibody 1:80	Proviral load 37%	Antibody 1:16384	Antibody present	Proviral load 108%
HTLV-1 CSF	Antibody 1:128	Antibody 1:1	Proviral load 52%	Antibody 1:8192	Antibody present	Proviral load 26%
Brain imaging	CT- cortical swelling and mild ventricular dilatation		Normal MR	MR-ADEM-like parieto-occipital white matter changes	MR-patchy hyper-intensity in cerebral white matter	Normal CT
EEG	–	**–**	Generalised slowing.	Polyspike and wave, then slow-wave bursts	Diffuse theta waves; paroxysmal delta and sharp waves	**–**
Treatment	Prednisolone and antibiotics	IV corticosteroids	Phenytoin	IV corticosteroids	IV corticosteroids	IV corticosteroids
Outcome	Died of pneumonia	Marked improvement	Died of pneumonia	Died	Ongoing myelopathy and polyneuropathy; resolution of cognitive symptoms	Died of pneumonia

Normal CSF ranges: white cells 0–5 per mm^3^, protein 15–45 mg/dL

*OP* opening pressure, *PBMC* peripheral blood mononuclear cells, *ADEM* acute disseminated encephalomyelitis

Eight of nine cases (including patients A–C described above) occurred in women. The mean patient age at the time of encephalopathy was 47 (range 13–73). One was < 20 years, four aged 30–50, and four aged > 50. Four were Japanese, two Afro-Caribbean, two White British, and one Brazilian. In the seven patients who also had HAM, the time between myelopathy onset and encephalopathy onset ranged from < 4 weeks to 24 years. In all patients with HAM, a sub-acute prodromal deterioration in mobility occurred. Five of nine cases had fever or hypothermia at encephalopathy onset [18, 21]. All had altered mental state and reduced conscious level. Four of nine had seizures [17–19].

Six of nine patients had a CSF pleocytosis [18, 19, 21], predominantly either monocytes or lymphocytes. CSF protein was high in six patients [18–20]. In all patients, HTLV-1 antibody was present in both serum and CSF. Where proviral load was measured, this was higher in CSF than PBMCs in three of four patients [17]. Where performed, MRI brain and EEG showed various abnormalities, with few consistent features across subjects, though three had reversible white matter changes on MRI. Of seven patients treated with IV corticosteroids [18–22], four responded well with resolution of encephalopathy.

Pathological findings were reported in three cases [17, 18, 21]. Perivascular CD8+ lymphocytic infiltrates were predominant in the brain and cord. Adjacent parenchymal CD8+ lymphocytic infiltration with microglial reaction and gliosis was also reported [18]. Cord demyelination was seen in all cases. No cerebral demyelinatinon was seen in either of the HAM cases biopsied. In one of the HAM cases, lymphocytic infiltrates were also present in multiple other organs, including skeletal muscle, liver, skin, salivary, adrenal and pituitary glands [18].

Two cases have also been reported of patients with NMO and HTLV-1 infection who developed treatment-refractory encephalopathy [15, 16]. One was treated with steroids, plasma exchange and rituximab, but remained blind, paraplegic and with impaired comprehension in the long term. The other NMO patient received methylprednisolone, plasma exchange, and methotrexate, but remained comatose.

## Discussion

In the UK, an underlying cause is not identified in more than a third of cases of acute encephalitis [23]. HTLV-1 is not widely recognised as a cause of encephalopathy and most articles on the neurology of HTLV-1 infection do not mention this as a possible manifestation. In our UK HAM cohort, there is a higher rate of observed encephalitis episodes than in the background population. To date, none of the 350 asymptomatic HTLV carriers in our cohort have had episodes of encephalopathy, suggesting that the immunological trigger for HAM is also important for the development of HTLV-associated encephalopathy. Where patients had an encephalomyelitis, the symptoms associated with spinal cord pathology worsened in the weeks prior to onset of encephalopathy.

One possibility is that HTLV is not the direct causative agent of the encephalopathy, but through CNS immunomodulation and effects on the blood brain barrier [24], infected individuals may be more susceptible to encephalopathy when other recognised risk factors are present. This mechanism might underlie the two case reports of severe and treatment-refractory NMO encephalopathy reviewed here. This compounding phenomenon is well recognised in co-infections such as strongyloidiasis, where the inflammation associated with HTLV infection leads to a hyperactive immune response to the parasite, causing much higher morbidity and mortality than is otherwise seen [25]. Nevertheless, in most patients discussed here, no other potential aetiological agent was identified, lending weight to the hypothesis that HTLV itself can cause encephalopathy, in the absence of co-existing infectious or autoimmune risk factors.

The majority of reported HTLV-associated encephalopathy has been in women. HTLV-associated myelopathy is also more common in women than in men. Furthermore, being female is a risk factor for developing a more aggressive tempo of myelopathy progression in HTLV infection [26]. Sexual dimorphism in immune phenotypes [27, 28] may underlie this gender skew, both in HAM and in HTLV-associated encephalopathy. HTLV-1 is associated with a broad range of presentations, all characterized by lymphocytic infiltrates in the affected tissue [29]. If HTLV-1 can cause encephalopathy, we suggest that this is as part of a spectrum of disease with HTLV-associated myelopathy.

## Conclusion

HTLV-1 infection should be considered a possible cause of encephalopathy, where no other aetiology is identified, particularly in patients from endemic areas or where there is co-existing myelopathy. HTLV-1 may also increase the risk of encephalopathy in patients with existing risk factors such as co-infections or autoimmune conditions. If HTLV-associated encephalopathy is suspected, IV corticosteroids may be an effective initial treatment option.

## Electronic supplementary material

Below is the link to the electronic supplementary material.

Supplementary material 1 (PPTX 37 kb)
