# Supplementary material for: Human T-cell lymphotropic virus (HTLV)-associated encephalopathy: an under-recognised cause of acute encephalitis? Case series and literature review
Source: J Neurol. 2018 Feb 8;265(4):871–9. doi: 10.1007/s00415-018-8777-z (PMC5878187; doi:10.1007/s00415-018-8777-z)
Supplement: Supplementary file 1 — Supplementary material 1 (PPTX 37 kb) [file 415_2018_8777_MOESM1_ESM.pptx]

## Slide 1
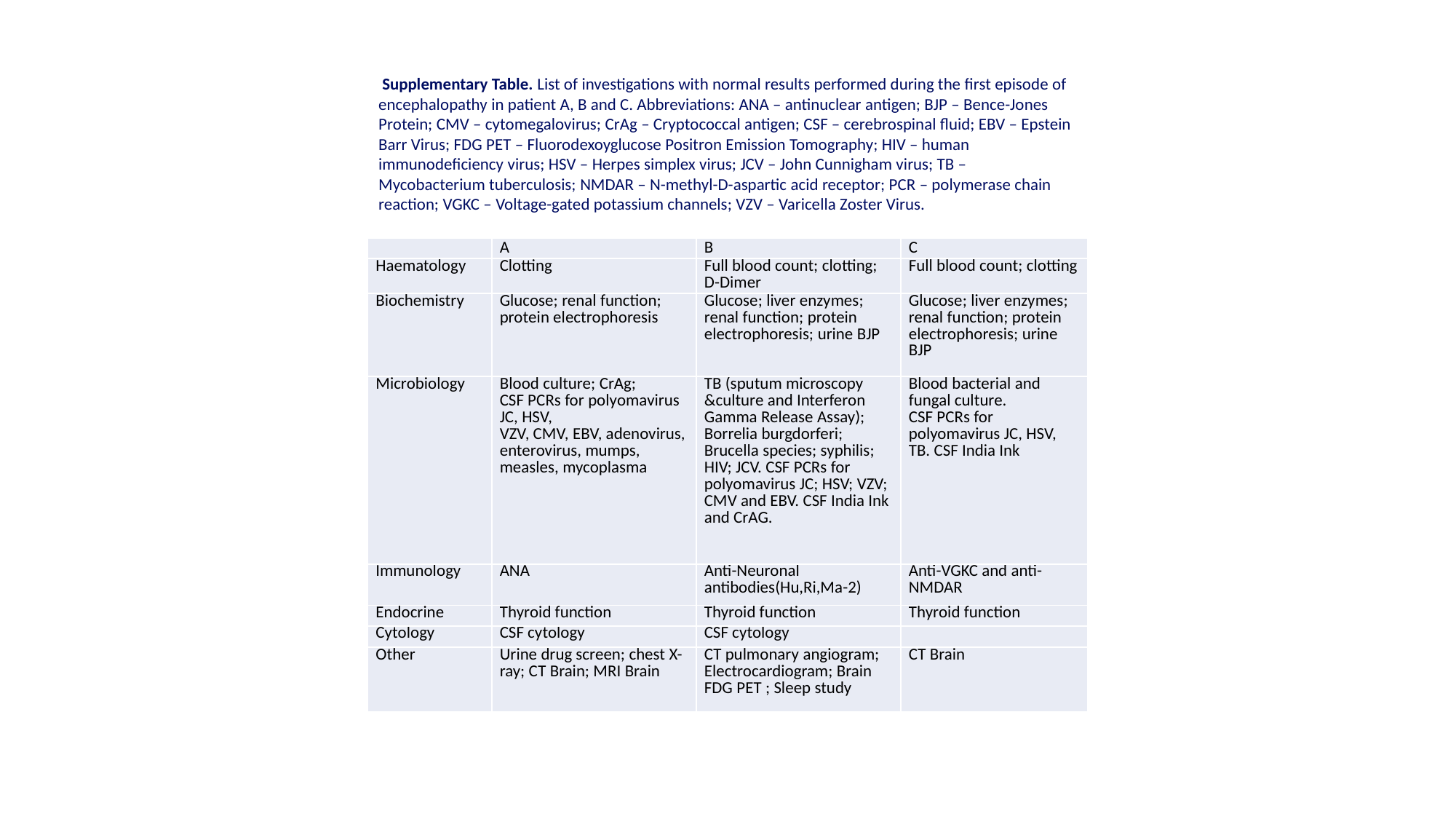

Supplementary Table. List of investigations with normal results performed during the first episode of encephalopathy in patient A, B and C. Abbreviations: ANA – antinuclear antigen; BJP – Bence-Jones Protein; CMV – cytomegalovirus; CrAg – Cryptococcal antigen; CSF – cerebrospinal fluid; EBV – Epstein Barr Virus; FDG PET – Fluorodexoyglucose Positron Emission Tomography; HIV – human immunodeficiency virus; HSV – Herpes simplex virus; JCV – John Cunnigham virus; TB – Mycobacterium tuberculosis; NMDAR – N-methyl-D-aspartic acid receptor; PCR – polymerase chain reaction; VGKC – Voltage-gated potassium channels; VZV – Varicella Zoster Virus.
| | A | B | C |
| --- | --- | --- | --- |
| Haematology | Clotting | Full blood count; clotting; D-Dimer | Full blood count; clotting |
| Biochemistry | Glucose; renal function; protein electrophoresis | Glucose; liver enzymes; renal function; protein electrophoresis; urine BJP | Glucose; liver enzymes; renal function; protein electrophoresis; urine BJP |
| Microbiology | Blood culture; CrAg; CSF PCRs for polyomavirus JC, HSV, VZV, CMV, EBV, adenovirus, enterovirus, mumps, measles, mycoplasma | TB (sputum microscopy &culture and Interferon Gamma Release Assay); Borrelia burgdorferi; Brucella species; syphilis; HIV; JCV. CSF PCRs for polyomavirus JC; HSV; VZV; CMV and EBV. CSF India Ink and CrAG. | Blood bacterial and fungal culture. CSF PCRs for polyomavirus JC, HSV, TB. CSF India Ink |
| Immunology | ANA | Anti-Neuronal antibodies(Hu,Ri,Ma-2) | Anti-VGKC and anti-NMDAR |
| Endocrine | Thyroid function | Thyroid function | Thyroid function |
| Cytology | CSF cytology | CSF cytology | |
| Other | Urine drug screen; chest X-ray; CT Brain; MRI Brain | CT pulmonary angiogram; Electrocardiogram; Brain FDG PET ; Sleep study | CT Brain |
